# miR-10a suppresses colorectal cancer metastasis by modulating the epithelial-to-mesenchymal transition and anoikis

**DOI:** 10.1038/cddis.2017.61

**Published:** 2017-04-06

**Authors:** Yankun Liu, Yingnan Zhang, Haidong Wu, Yufeng Li, Yi Zhang, Min Liu, Xin Li, Hua Tang

**Affiliations:** 1 Tianjin Life Science Research Center, Department of Pathogen Biology, School of Basic Medical Sciences, Tianjin Medical University, Tianjin, China; 2 The Central Laboratory of Cancer Institute, Tangshan People's Hospital, Tangshan, China

## Abstract

MicroRNAs (miRNAs) have a critical role in tumorigenesis and metastasis, which are major obstacles of cancer therapy. However, the role of miRNAs in colorectal cancer (CRC) metastasis remains poorly understood. Here, we found that miRNA-10a (miR-10a) was upregulated in primary CRC tissues and cell line (SW480) derived from primary CRC compared with metastatic cancer tissues in lymph node and cell line (SW620). The differential expression of miR-10a was inversely correlated with distant metastasis and invasion depth. miR-10a promoted migration and invasion *in vitro* but inhibited metastasis *in vivo* by regulating the epithelial-to-mesenchymal transition and anoikis. Furthermore, matrix metalloproteinase 14 (MMP14) and actin gamma 1 (ACTG1) were validated as target genes of miR-10a in CRC cells. Ectopic expression of MMP14 and ACTG1 counteracted the decreased cell adhesion and anoikis resistance activities induced by miR-10a. These findings not only describe the mechanism by which miR-10a suppresses CRC metastasis but also suggest the potential prognostic and therapeutic value of miR-10a in CRC patients.

## 

Colorectal cancer (CRC) is the third most common cancer worldwide and is the fourth leading cause of cancer-related deaths.^1, 2^ Local recurrence and distant metastasis remain major causes of CRC-related death.^3^ Metastasis can be simply portrayed as a two-phase cascade process: the physical translocation of a cancer cell from the primary tumor to the microenvironment of a distant tissue, followed by colonization. The epithelial-to-mesenchymal transition (EMT) is the first event involved in tumor progression. During the EMT, basal epithelial cells lose the 'epithelial phenotype', leading to a loss of apical–basal polarity. These cells subsequently acquire the 'mesenchymal phenotype'. The features of these cells, including epithelial marker (e.g., E-cadherin) downregulation, mesenchymal marker (e.g., vimentin) upregulation and extracellular matrix (ECM) disruption, will trigger 'anoikis'.^4, 5^ Anoikis occurring in detached cells can prevent them from reattaching to inappropriate matrices and resuming growth. Particularly, anoikis resistance in cancer cells allows anchorage-independent growth, which has a crucial role in the second phase of tumor metastasis.^6^ However, the mechanisms of the cascading process of CRC metastasis regulated by the EMT and anoikis are not well understood.

microRNAs (miRNAs) constitute an evolutionarily conserved class of pleiotropically acting small RNAs that suppress gene expression post-transcriptionally via sequence-specific interactions with the 3' untranslated region (3'UTR) of cognate mRNA targets^7^ or promote gene expression by binding to mRNA 3'UTR in a G-rich RNA sequence binding factor 1 (GRSF1)-dependent manner.^8^ They are extensively involved in many biological processes, such as cell proliferation, differentiation, metabolism and apoptosis.^9, 10^ miRNA dysregulation has been shown to contribute to tumor initiation, progression and metastasis.^11, 12^ Some miRNAs function as oncogenes or tumor-suppressor genes, which may regulate tumor invasion- and metastasis-related processes, such as the EMT^13, 14, 15, 16^ or anoikis.^17, 18^ A few studies have focused on the role of miRNAs in the metastasis cascade step following local invasion in hepatocellular carcinoma cells^19^ and gastric cancer (GC) cells.^20^ Currently, the extent to which miRNAs are involved in this critical step during CRC metastasis remains unclear.

In this study, we identified the miRNAs expressed differently in SW480 and SW620 cell lines, which were separately isolated from the same CRC patient with primary site (SW480 cells) in the early phase and metastatic cancer loci (SW620 cells) in a lymph node that developed months later.^21^ We focused on miRNA-10a (miR-10a), which was more abundant in SW480 cells than in SW620 cells. We analyzed the correlation of miR-10a expression with CRC clinical parameters, migration and invasion *in vitro*, and metastasis *in vivo*, as well as its role in regulating the EMT and CRC cells migration, adhesion and anoikis. Furthermore, we validated its functional target genes and clarified their roles in CRC metastasis.

## Results

### SW480 cells exhibit stronger migration activity but weaker cell adhesion and anoikis resistance than SW620 cells

**Figure 1:**
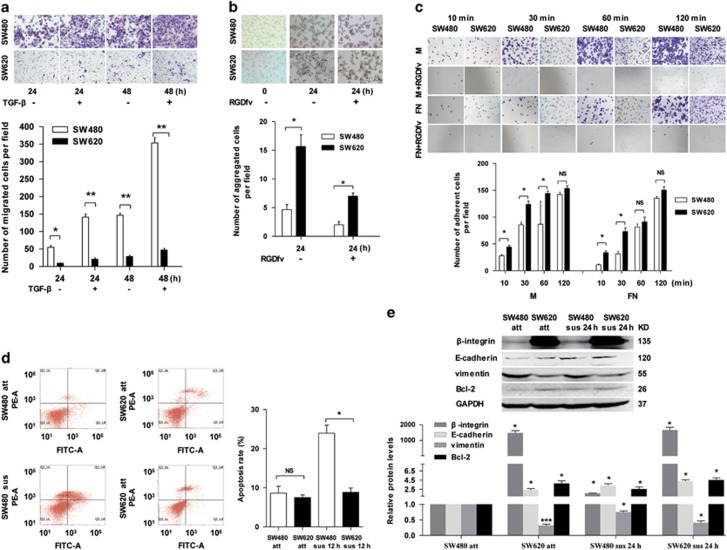
SW480 cells exhibit stronger migration activity but weaker cell adhesion and anoikis resistance than SW620 cells. (**a**) Transwell migration assay of SW480 and SW620 cells were seeded and cultured in the Boyden chamber well with or without TGF-*β* for 24 and 48 h. Above: representative images. Below: quantitative results of three independent experiments (*P<0.05, **P<0.01). (**b**) Cell–cell adhesion assay of SW480 and SW620 cells suspension cultured with or without cell adhesion inhibitor (RGDfv) for 24 h. Above: representative images, the photomicrographs were taken at × 100 magnification. Below: quantitative results of three independent experiments (*P<0.05). (**c**) Cell–matrix adhesion assay of SW480 and SW620 cells adhering to FN and Matrigel (M) with or without RGDfv for 10, 30, 60 and 120 min, respectively. Above: representative images (100 × ). Below: quantitative results of three independent experiments. The adherent cells were measured by software of ImageJ (NS indicates no statistical difference between the groups, *P<0.05). (**d**) FACS analysis of the apoptosis of attached/suspended (att/sus) SW480 and att/sus SW620 cells cultured for 12 h. Left: representative images. Right: quantitative results of three independent experiments (NS indicates no statistical difference between the groups, *P<0.05). (**e**) Western blot results showing E-cadherin, vimentin, *β*-integrin and Bcl-2 expression in SW480 and SW620 cells with attached (att) and suspended (sus) growth manner. The expression levels were normalized to GAPDH (*P<0.05, ***P<0.001)

As SW480 and SW620 cell lines were isolated from CRC primary site (SW480) and metastatic cancer loci (SW620) in lymph node of the same patient. We observed substantial differences in morphology with the spindle- and fibroblast-like shape in the SW480 cells and the cobble-stone-like appearance for SW620 cells (Supplementary Figure S1). To determine the differences of metastasis-related malignant phenotypes in the SW480 and SW620 cells, experiments of migration/invasion and anoikis were performed. Compared with SW620 cells, the SW480 cells exhibited significantly increased migration activity by migration assay with or without transforming growth factor (TGF)-*β* induced for different time (Figure 1a). In contrast, the SW480 cells were less aggregated than SW620 cells suspension cultured with or without cell adhesion inhibitor RGDfv (Figure 1b), less adhesion to fibronectin (FN) and Matrigel (Figure 1c), and weaker resistant to anoikis than SW620 cells (Figure 1d). Furthermore, the level of the mesenchymal marker vimentin was significantly higher in SW480 cells, whereas the levels of the epithelial marker E-cadherin, the cell adhesion molecule *β*-integrin and the anti-apoptosis protein Bcl-2 were markedly lower than those in SW620 cells with attachment (att) or suspension (sus) growth manner (Figure 1e,Supplementary Figure S4). These results indicated that there are differences of EMT and anoikis in SW480 and SW620 cells, which suggested the differently metastatic properties of SW480 and SW620 cells in CRC metastasis.

### miR-10a is upregulated in primary CRC cells and tissues

**Figure 2:**
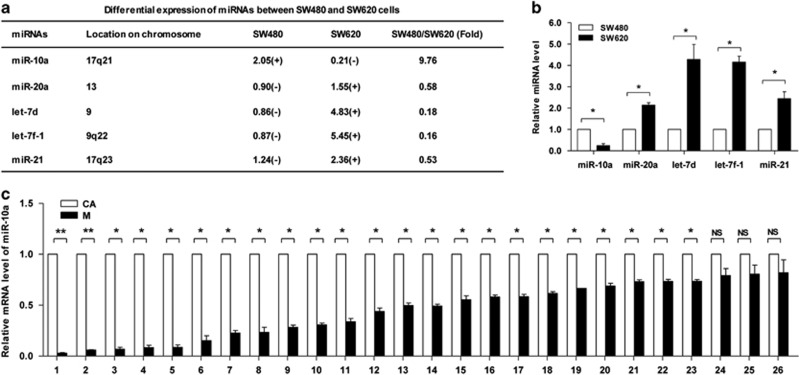
miR-10a is overexpressed in primary CRC cells and tissues. (**a**) The miRNAs differentially expressed between SW480 and SW620 cells were analyzed using a miRNA microarray. The five miRNAs exhibiting the significant differences are shown. The miRNAs with a signal background ratio of 1.5 or greater were considered to represent positive expression (+), and the others were considered to represent negative expression (−). (**b**) RT-qPCR results of the five miRNAs in SW480 and SW620 cells (*P<0.05). (**c**) RT-qPCR results of miR-10a in human primary CRC tissues (CA) and paired lymph node metastases (M). The expression levels were normalized to U6 snRNA (*P<0.05, **P<0.01, NS, not significant)

To identify the specific miRNAs involved in CRC metastasis, a human miRNA microarray with 328 probes was used to assess miRNA expression in SW480 cells and SW620 cells. The results showed that miR-20a, let-7d, let-7 f-1 and miR-21 were predominantly upregulated in SW620 cells, but miR-10a was significantly upregulated in SW480 cells. The relative level of miR-10a in SW480 cells was 8.76-fold greater than that in SW620 cells, markedly higher than the other four miRNAs (Figure 2a). The differential expression of these five miRNAs in SW480 and SW620 cells was confirmed using stem-loop reverse-transcription quantitative PCR (RT-qPCR) (Figure 2b). Among these five altered miRNAs, only miR-10a was upregulated in SW620 cells. We speculated that miR-10a may have a crucial role in CRC metastasis.

Next, we analyzed miR-10a level in 26 pairs of clinical formalin-fixed, paraffin-embedded (FFPE) samples (CRC primary site and metastatic cancer in paired lymph node); miR-10a was highly expressed in 23 (88.5%) CRC primary site compared with metastatic cancer in lymph node (Figure 2c). Given that miR-10a could promote human non-small cell lung carcinoma^22^ and cervical cancer^23^ cells proliferation, migration and invasion, the associations between miR-10a levels and clinicopathological data were analyzed. Result indicated that the relative expression of miR-10a in the primary site of CRC tissues compared with metastatic cancer in correspondence lymph node was inversely correlated with distant metastasis and invasion depth (Supplementary Table S1), which suggested that miR-10a may be a potential negative regulator of CRC metastasis.

### miR-10a promotes the invasion and suppresses the adhesion and anoikis resistance of CRC cells *in vitro* but suppresses metastasis *in vivo*

**Figure 3:**
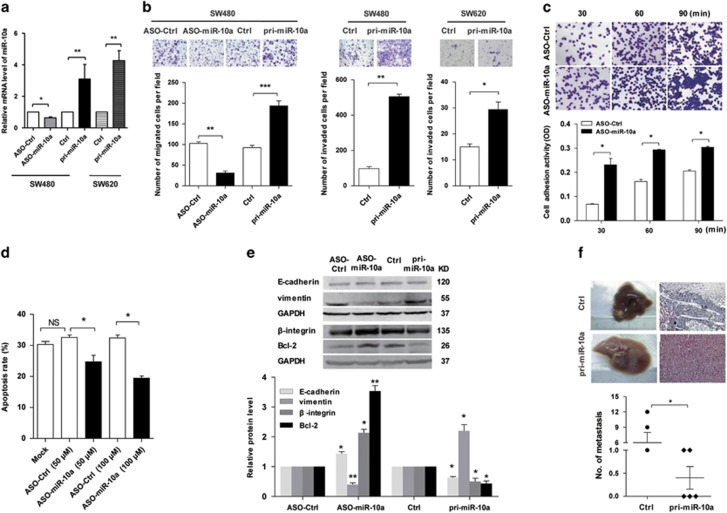
miR-10a promotes the invasion and suppresses the adhesion and anoikis resistance of CRC cells *in vitro* but suppresses metastasis *in vivo*. (**a**) RT-qPCR assay of the miR-10a expression in the indicated groups of SW480 and SW620 cells (*P<0.05, **P<0.01). (**b**) Transwell migration assay of SW480 cells transfected with ASO-miR-10a or control oligonucleotides (ASO-Ctrl) and pri-miR-10a or control vector (Ctrl), and transwell invasion assay of SW480 and SW620 cells transfected with pri-miR-10a. Cells in five random fields of view at × 100 and × 200 magnification for SW480 cells and SW620 cells were counted, and the representative images are indicated (**P<0.01, **P<0.01, ***P<0.001). (**c**) Cell–matrix adhesion assay of SW480 cells with silenced miR-10a expression (*P<0.05). (**d**) FACS assay for measuring apoptosis of SW480 cells transfected with different concentrations of ASO-miR-10a in suspension culture 48 h (*P<0.05, NS, not significant versus Mock). (**e**) Western blot results for *β*-integrin, Bcl-2, E-cadherin and vimentin in the indicated SW620 cells. The expression levels were normalized to GAPDH (*P<0.05, **P<0.01). (**f**) *In vivo* metastasis assay. Upper: representative livers and the metastatic nodules from spleens injected with SW620 cells are indicated. Representative H&E staining results of metastatic nodules in the liver are shown. Lower: the statistical results of the metastatic nodules are indicated (*n*=5, *P<0.05)

To determine whether miR-10a is involved in proliferation and invasion in CRC cells, miR-10a was silenced or overexpressed in SW480 cells and SW620 cells by transfection with ASO-miR-10a or pri-miR-10a, respectively. Compared with the control groups (ASO-Ctrl), miR-10a level of SW480 cells in ASO-miR-10a group was reduced by approximately 35.67%. Although miR-10a levels of SW480 and SW620 cells in pri-miR-10a group were enhanced by approximately 22.6- and 3.26-folds, respectively (Figure 3a). The CCK-8 assay indicated that the altered levels of miR-10a did not significantly affect SW480 or SW620 cell viability and proliferation (Supplementary Figure S2). Conversely, pri-miR-10a markedly increased and ASO-miR-10a significantly decreased the migration of SW480 cells, and miR-10a overexpression enhanced the invasion of both SW480 and SW620 cells (Figure 3b). Furthermore, we explored whether miR-10a regulates CRC cell adhesion. The results showed that ASO-miR-10a enhanced cell–matrix adhesion by approximately 240.0%, 80.9% and 48.3% at 30, 60 and 90 min, respectively (Figure 3c). In addition, ASO-miR-10a increased the anoikis resistance of SW480 cells with suspension culture for 48 h (Figure 3d) but did not affect cell viability (Supplementary Figure S3). Next, we found that ASO-miR-10a upregulated E-cadherin, *β*-integrin and Bcl-2 but downregulated vimentin in SW480 cells, whereas pri-miR-10a exerted the opposite effects (Figure 3e,Supplementary Figure S5). These results suggested that miR-10a promoted the EMT and anoikis in SW480 cells *in vitro*.

To further investigate whether miR-10a could influence the process of metastasis *in vivo*, a xenograft model in nude mice was applied. Control cells or SW620 cells overexpressing miR-10a were transplanted into the upper pole of the spleen of nude mice. After 6 or 8 weeks, the spleens and livers were harvested. Surprisingly, most mice (4/5) in the control group had developed liver metastases, and two mice bore >2 metastatic nodules. However, only two of the five mice in the pri-miR-10a group generated liver metastases, and both the number of metastatic nodes and tumor size were less than those in the control group (Figure 3f). These results indicated that miR-10a suppressed SW620 cell liver metastasis *in vivo*, which contradicted the function of miR-10a observed *in vitro* because the promotion of migration and invasion *in vitro* is usually considered to represent the potential for cancer metastasis *in vivo*.

### miR-10a directly targets the 3'-UTR of MMP14 and ACTG1 transcripts

**Figure 4:**
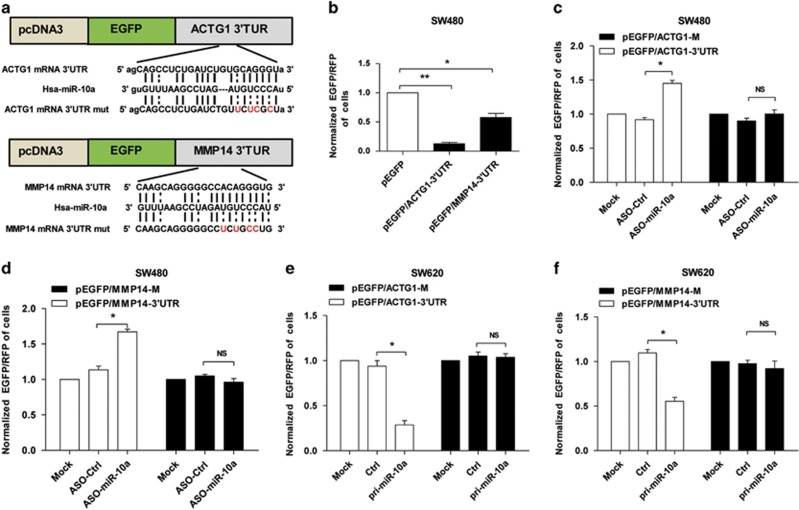
miR-10a directly targets the 3′-UTR of MMP14 and ACTG1 transcripts. (**a**) The predicted binding sites for miR-10a in the 3′-UTR of MMP14 and ACTG1 mRNAs, and the mutations as indicated. (**b**) The EGFP reporter assay was performed in SW480 and SW620 cells transfected with reporter vectors (*P<0.05, **P<0.01). (**c** and **d**) EGFP intensity was detected in SW480 cells with blocked miR-10a (*P<0.05, NS, not significant). (**e** and **f**) EGFP intensity was detected in miR-10a overexpressing SW620 cells (all results performed above are presented as mean±S.D. from three independent experiments, *P<0.05, NS, not significant)

To explore the functional targets of miR-10a in tumor metastasis, bioinfomatics predicted that the 3′-UTR of matrix metalloproteinase 14 (MMP14) and actin gamma 1 (ACTG1) transcripts contain miR-10a-binding sites (Figure 4a). Therefore, we chose MMP14 and ACTG1 for further study with consideration of functional knowledge. The EGFP reporter assay showed that the fluorescence intensity of SW480 cells transfected with vectors containing the 3′-UTR of MMP14 or ACTG1 was significantly decreased compared with the control, which indicated that endogenous miR-10a negatively regulated MMP14 and ACTG1 in SW480 cells (Figure 4b). Furthermore, the fluorescence intensity was reduced or enhanced according to the overexpression or silencing of miR-10a but was not affected when the reporter vectors carried the mutant 3′-UTRs (Figure 4a) within the miR-10a-binding sites (Figures 4c–f). These data indicated that miR-10a directly targeted the 3′-UTR of MMP14 and ACTG1 and may suppress their expression.

### miR-10a negatively regulates MMP14 and ACTG1 expression at the mRNA and protein levels in CRC cells and tissues

**Figure 5:**
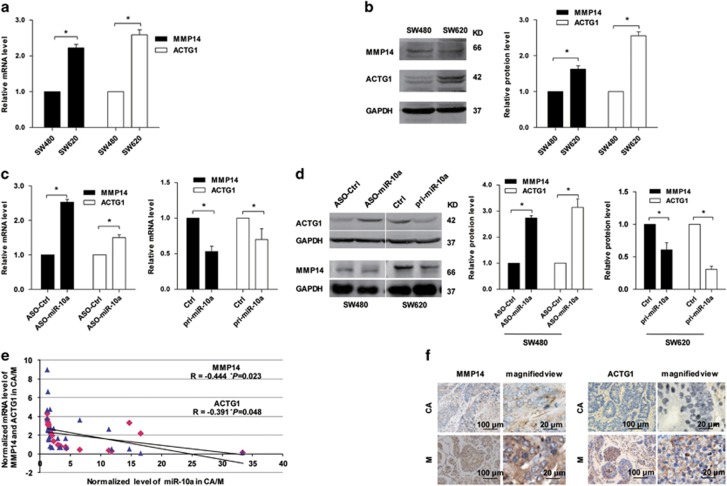
miR-10a negatively regulates the expression levels of MMP14 and ACTG1. (**a** and **b**) The mRNA and protein levels of MMP14 and ACTG1 in SW480 and SW620 cells were detected. The mRNA level was normalized to *β*-actin and the protein level was normalized to GAPDH. The data represented three measurements. Results are expressed as mean±S.D. *P<0.05. (**c**) The mRNA levels of MMP14 and ACTG1 in SW480 cells transfected with ASO-miR-10a or ASO-Ctrl and pri-miR-10a or Ctrl were detected. Results are expressed as mean±S.D. *P<0.05. (**d**) The protein levels of MMP14 and ACTG1 in SW480 cells transfected with ASO-miR-10a or ASO-Ctrl and SW620 cells transfected with pri-miR-10a or Ctrl were examined. Results are expressed as mean±S.D. *P<0.05. (**e**) Pearson's correlation analysis of the negative correlation between the expression of miR-10a and MMP14/ACTG1 (*P<0.05). (**f**) Representative IHC images of MMP14 and ACTG1 in primary CRC tissues and paired lymph node metastases are shown

Next, we analyzed the effect of miR-10a on MMP14 and ACTG1 expression. Both the mRNA and protein levels of MMP14 and ACTG1 were markedly higher in SW620 cells than those in SW480 cells (Figures 5a and b,Supplementary Figure S6), which were inversely correlated with miR-10a expression. Furthermore, the expression levels of MMP14 and ACTG1 in SW480 cells after ASO-miR-10a transfection were enhanced at both the mRNA and protein levels (Figures 5c and d,Supplementary Figure S6). The mRNA levels of MMP14 and ACTG1 in 26 CRC specimens were inversely correlated with the downregulation (Figure 2c) of miR-10a (Figure 5e). Furthermore, the protein levels of MMP14 and ACTG1 in the CRC samples were detected by immunohistochemistry (IHC). The observations suggested that the MMP14 and ACTG1 protein levels were enhanced in CRC metastastic tissues in lymph node (Figure 5f) and were inversely correlated with miR-10a levels. These results revealed that MMP14 and ACTG1 were downregulated by miR-10a in CRC cells and tissues.

### MMP14 and ACTG1 overexpression enhances the migration and adhesion induced by *β*-integrin in SW480 cells

**Figure 6:**
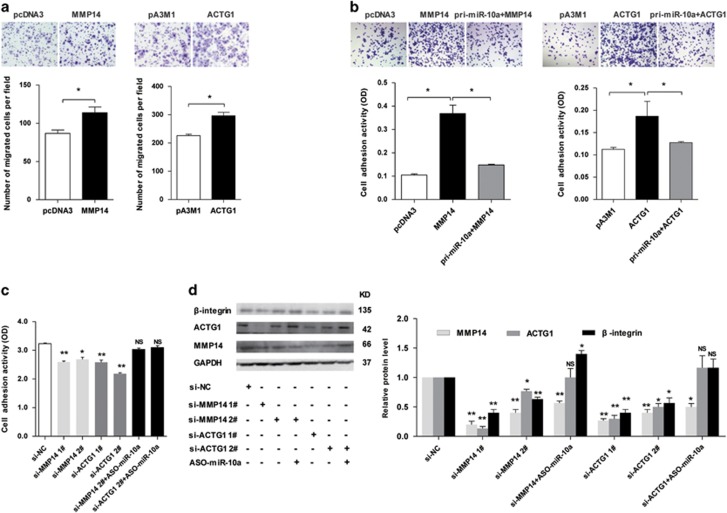
MMP14 and ACTG1 overexpression enhances SW480 cell migration and adhesion by upregulating *β*-integrin. (**a**) Transwell migration assay of SW480 cells with overexpressing MMP14 and ACTG1. Above: representative images, the photomicrographs were taken at 100 × magnification. Below: quantitative results of three independent experiments (*P<0.05). (**b**) Adhesion assay of SW480 cells with overexpressing MMP14 and ACTG1. The number of adherent cells was estimated by reading the absorbance at 620 nm. Above: representative images, the photomicrographs were taken at × 100 magnification. Below: quantitative results of three independent experiments (*P<0.05). (**c**) Adhesion assay of SW480 cells transfected with the indicated siRNAs (*P<0.05, **P<0.01, NS, not significant). (**d**) The influence of MMP14 and ACTG1 on *β*-integrin protein expression in SW480 cells was determined by western blot (*P<0.05, **P<0.01, NS, not significant)

To address the metastasis-associated features of CRC cells regulated by MMP14 and ACTG1, pcDNA3-ACTG1-myc (ACTG1) and pcDNA3-MMP14 (MMP14) plasmids were constructed to intensify MMP14 and ACTG1 expression, and si-MMP14 1#, si-MMP14 2#, si-ACTG1 1# and si-ACTG1 2# siRNAs were synthesized to silence the endogenous MMP14 and ACTG1. The migration assay showed that both MMP14 and ACTG1 overexpression significantly enhanced SW480 cell migration (Figure 6a). The adhesion assay indicated that adhesion activities of MMP14- and ACTG1-overexpressed SW480 cells were, respectively, enhanced by approximately 250% and 56.6% compared with the control groups, whereas miR-10a overexpression counteracted the promotion of cell adhesion (Figure 6b). In addition, SW480 cells transfected with the MMP14 and ACTG1 siRNAs attenuated cell–matrix adhesion, and this suppressed adhesion induced by ACTG1 and MMP14 was restored in cells co-transfected with ASO-miR-10a (Figure 6c).

Integrins is hemidesmosomes allow basal adhesion,^24, 25^ and their inhibition has been shown to repress the EMT and metastasis in triple-negative breast cancer cells,^26^ as well as attenuate melanoma cell migration by inducing anoikis.^27^ We assessed the influence of miR-10a and its targets, MMP14 and ACTG1, on integrins. As shown in Figure 6d, SW480 cells with suppressed MMP14 and ACTG1 expression exhibited less *β*-integrin compared with the control group. In contrast, the western blot assay showed that the reduced *β*-integrin level was rescued in cells co-transfected with ASO-miR-10a (Figure 6d, Supplementary Figure S7). These results revealed that MMP14 and ACTG1 overexpression enhanced cell migration and adhesion, and MMP14 and ACTG1 suppression decreased cell adhesion via *β*-integrin.

### MMP14 and ACTG1 promote SW620 cell resistance to anoikis

**Figure 7:**
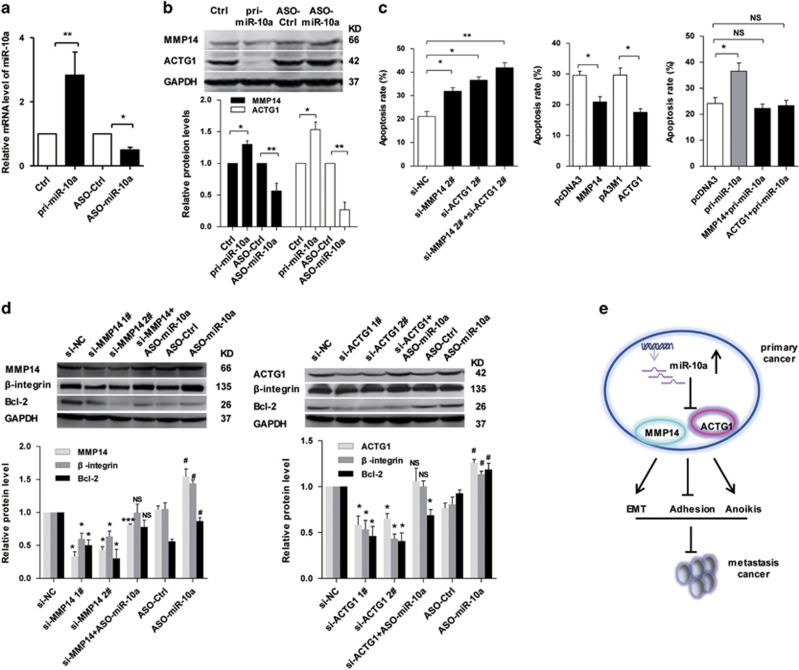
MMP14 and ACTG1 promotes the anoikis resistance of SW620 cells by upregulating Bcl-2. (**a**) RT-qPCR assay of miR-10a levels in SW620 cells transfected with pri-miR-10a and ASO-miR-10a compared with the control groups (*P<0.05, **P<0.01). (**b**) The protein levels of MMP14 and ACTG1 induced by pri-miR-10a and ASO-miR-10a in SW620 cells. Represent images and histogram were showed. The data represented three measurements (*P<0.05, **P<0.01). (**c**) FACS results of the apoptosis of SW620 cells in each group, as indicted. Quantitative results of three independent experiments are shown (*P<0.05, **P<0.01, NS, not significant). (**d**) Western blot results for *β*-integrin, Bcl-2, MMP14 and ACTG1 in the indicated SW620 cells. The data represented three measurements. Results are expressed as mean±S.D. (*P<0.05 and **P<0.01 versus si-NC group, ^#^*P*<0.05 versus ASO-Ctrl group, NS, not significant versus si-NC group). (**e**) Schematic describing the roles of MMP14 and ACTG1 as regulated by miR-10a in CRC metastasis

To assess the functional roles of MMP14 and ACTG1 mediated by miR-10a in anoikis in CRC cells, the pri-miR-10a and ASO-miR-10a were used to enhance and reduce the miR-10a expression levels in SW620 cells (Figure 7a). Then, we examined the protein levels of MMP14 and ACTG1 in SW620 cells were induced by miR-10a. As shown in Figure 7b and Supplementary Figure S8, the MMP14 and ACTG1 were decreased in the pri-miR-10a group but increased in the ASO-miR-10a group of SW620 cells, respectively. To examine the effect on anoikis by MMP14 and ACTG1 in SWW620 cells, MMP14 and ACTG1 expression levels were silenced using synthesized siRNAs and enhanced by transfection with the plasmids of ACTG1 and MMP14, respectively. Compared with the control group, MMP14 and ACTG1 knockdown enhanced anoikis by approximately 47.6% and 69.6% in SW620 cells, respectively, whereas knockdown of MMP14 and ACTG1 enhanced anoikis by 90.1% in SW620 cells. Moreover, ACTG1 and MMP14 overexpression repressed anoikis by approximately 25.3% and 37.6% in SW620 cells compared with the control group, respectively. Consistent with the results that miR-10a promoted anoikis as shown in Figure 3d, MMP14 and ACTG1 overexpression counteracted the promotion of anoikis induced by miR-10a (Figure 7c).

Bcl-2 is recognized as a marker of the intrinsic pathway of anoikis.^28^ Thus, we investigated whether Bcl-2 is involved in CRC cell anoikis regulated by miR-10a, MMP14 and ACTG1. Western blot assay demonstrated that the protein level of Bcl-2 was actually decreased when MMP14 and ACTG1 silenced compared with the control group, whereas the depletion of miR-10a actually rescued the decreased Bcl-2 levels, as well as *β*-integrin (Figure 7d,Supplementary Figure S8). These results revealed that MMP14 and ACTG1 may mediate regulation of miR-10a on resistance to anoikis in CRC cells. Together, these results indicated that miR-10a inhibited adhesion to initiate the EMT but promoted anoikis to suppress metastasis by downregulating MMP14 and ACTG1 expression (Figure 7e).

## Discussion

Cancer metastasis is defined by distinct steps involving local invasion, intravasation, dissemination in the circulation, extravasation, colonization and population to form micro- to macrometastatic foci.^29^ miRNAs have been reported to participate in each process of metastasis, either promoting or suppressing metastasis. In this study, we revealed that miR-10a promoted the EMT in CRC cells but suppressed adhesion and anoikis resistance *in vitro* in addition to repressing metastasis *in vivo* by targeting MMP14 and ACTG1.

To avoid genetic heterogeneity, SW480 cells (primary) and SW620 cells (metastatic) originating from the same patient were selected and used as an ideal model for studying CRC metastasis. In fact, SW480 and SW620 cells, which from different sub-populations, have been described within different membrane protrusions, surface roughness and skeletonized actin that affect cell migration and adhesion activities.^30^ In our study, SW480 cells with lower levels of E-cadherin and *β*-integrin exhibited more motility, less cell–cell and cell–matrix adhesion and more anoikis resistance with respect to SW620 cells. These results illustrate that SW480 cells in primary tumor facilitate to detach from their neighbors and invade the ECM and intravasate into the circulation, whereas the colonization in distant organs would likely be weak because of their sensitivity to anoikis and poor adhesion activity.

To determine whether miRNAs are involved in these processes, we used a miRNA array to profile the miRNAs differentially expressed between SW480 and SW620 cells. Among the 328 miRNAs detected, five miRNAs exhibited significant differences in expression, and only miR-10a was highly expressed in SW480 cells. Therefore, we focused on the regulation of miR-10a in CRC metastasis in this study. Most importantly, its level in the primary cancer compared with metastasis was inversely correlated with metastatic potency and clinical parameters. Thus, we hypothesized that miR-10a may be a negative regulator of CRC metastasis. Interestingly, when the causal effect of miR-10a and CRC metastasis was evaluated, miR-10a was found to enhance the migration and invasion of SW480 and SW620 cells by regulating the EMT *in vitro*, which seemed to contradict our speculation. In contrast, the intrasplenic injection of miR-10a-overexpressing SW620 cells suppressed the formation of liver metastases in nude mice. Therefore, miR-10a promotes migration and invasion *in vitro* but suppresses metastasis *in vivo*. These contradicting phenomena can be interpreted by two effects that miR-10a exerts in the cascading process of CRC metastasis. First, miR-10a promotes SW480 cells to detach from each other, disrupt the ECM, and migrate into vessels in the primary site. Second, miR-10a reduces cell adhesion and anoikis resistance, which prevents cells from surviving and colonizing in distant organs. Coincidentally, our previous studies demonstrated that miR-10a promoted QGY-7703 and HepG2 cell migration and invasion *in vitro* but inhibited their metastasis *in vivo*.^20^ However, a recent report showed that miR-10a promoted the development of GC and was overexpressed in lymph node metastasis cells.^31^ In addition, miR-10b, which is closely related to miR-10a, has been found to be highly expressed in metastatic breast cancer cells and positively regulates breast cancer cell migration, invasion and metastasis.^32^ miR-10a regulates metastasis in various cancer types, but the specific mechanism by which miR-10a regulates CRC metastasis may be different.

To further analyze the mechanisms by which miR-10a regulates CRC metastasis, the determination of its target genes is essential. Although cell adhesion molecule L1 (CHL1) and EphA4 have previously been identified as targets of miR-10a,^20, 23^ based on our bioinformatics analysis and knowledge of miR-10a function, we were the first to validate MMP14 (refs 33, 34) and ACTG1 (ref. 35) as EMT-related functional targets of miR-10a. There were several lines of evidence indicating that both MMP14 and ACTG1 were targets of miR-10a. First, miR-10a was overexpressed in SW480 cells, which resulted in the downregulation of MMP14 and ACTG1. Second, the EGFP reporter assay indicated that miR-10a could bind to the 3′-UTR of MMP14 and ACTG1 transcripts. Third, miR-10a overexpression or depletion significantly decreased or increased the expression of MMP14 and ACTG1 at the mRNA and protein levels in both CRC cells and tissues. These results indicated that miR-10a negatively regulated MMP14 and ACTG1 in CRC.

MMP14, an ECM remodeling protein, contributes to invasion, metastasis and angiogenesis through ECM degradation.^33, 34^ Another target gene of miR-10a, ACTG1, is involved in muscle contraction, cell motility, cell adhesion and cell shape maintenance.^35^ MMP14 and ACTG1 are important cell adhesion molecules involved in the EMT process. During the EMT program, epithelial cells undergo multiple biochemical changes, such as cell–cell adhesion disruption, marked cytoskeleton remodeling and mesenchymal characteristic acquisition.^36^ Silencing MMP14 or ACTG1 inhibited SW480 cell migration, adhesion and anoikis resistance. Integrin-mediated cell attachment has been shown to be required for cancer cell migration and metastasis.^37^ Moreover, *β*-integrin has been reported to function with MMP14, contributing to mammary epithelial cell invasion.^38^ We found that *β*-integrin was concomitantly decreased when MMP14 or ACTG1 was silenced in SW480 cells. In addition, migration could be inhibited by miR-10a silencing. Furthermore, cell adhesion was enhanced when MMP14 and ACTG1 were overexpressed, and miR-10a overexpression could counteract the enhanced adhesion in SW480 cells. Therefore, the downregulation of *β*-integrin, MMP14 and ACTG1 by miR-10a in SW480 cells caused the cells to detach from each other, thereby facilitating the initiation of the EMT process.

Integrins sense mechanical forces arising from the matrix and convert these stimuli into downstream signals that modulate cell viability. *β*-Integrin attenuates melanoma A375 and A2058 cell migration by inducing anoikis.^27^ Based on these observations, *β*-integrin downregulation may influence CRC cell survival during metastasis. The results showed that MMP14 and ACTG1 silencing resulted in SW620 cells becoming sensitive to anoikis and exhibiting reduced Bcl-2 expression. Whereas silencing miR-10a could reduce apoptosis and rescue Bcl-2 expression. These results suggest that MMP14 and ACTG1 are negatively regulated by miR-10a and promote SW620 cell resistance to anoikis during metastasis.

Collectively, these findings indicate that miR-10a downregulates MMP14 and ACTG1 to promote the EMT and anoikis, inhibiting CRC metastasis. Higher miR-10a levels are significantly correlated with less aggressive CRC phenotypes in patients. Our findings may shed light on a potential molecular mechanism by which this miRNA regulates CRC metastasis, which may have prognostic and therapeutic value for CRC patients in the future.

## Materials and Methods

### Human colon cancer samples

This study was approved by the ethics committee of the Cancer Institute of Tangshan People's Hospital. Written informed consent was obtained. As miRNAs are generally well preserved in a wide range of specimen types, including bodily fluids and FFPE tissues,^39^ a subset of 26 pairs of FFPE specimens (primary CRC tissues and lymph node metastasis tissues) from the same patient who had not received chemotherapy or radiotherapy were used to detect miR-10a expression. The metastatic activity of the CRC samples was confirmed according to the TNM classification.^40^ The specimens were sliced into 5-*μ*m sections, stained with hematoxylin and eosin (H&E) and evaluated for the most representative areas by a pathologist (median tumor content in the sample was >90%). Based on the diagnosis, the representative FFPE specimens (primary cancer site and metastatic cancer in the paired lymph node) were then enucleated and sliced into 5-*μ*m sections using a cryostat microtome and store at –80 °C until RNA isolation.

### Cell culture and transfection

SW480 and SW620 cells were isolated from primary CRC in the early phase and a lymph node metastasis that developed months later in the same patient. These cells were cultured in minimum essential medium-a (Gibco BRL, Grand Island, NY, USA) containing 10% fetal bovine serum (Gibco, BRL), 100 IU/ml of penicillin and 100 *μ*g/ml of streptomycin. The cell lines were incubated at 37 °C in a humidified incubator supplemented with 5% CO_2_. Transfection was performed with Lipofectamine 2000 (Invitrogen, Carlsbad, CA, USA) according to the manufacturer's protocol.

### miRNA microarray analysis

The miRNA microarray analysis was performed using a mirVana miRNA Probe Set and a mirVana miRNA Labeling Kit (Ambion, Austin, TX, USA) according to the manufacturer's instructions. The Cy3- or Cy5-labeled SW480 and SW620 RNA samples were hybridized to the microarray, which contained human miRNA probes in duplicate. The slides were scanned with a Packard Biochip Technologies ScanArray Express microarray acquisition system (PerkinElmer, Boston, MA, USA), and the scanned images were analyzed using ScanArray Express version 1.0. The miRNAs with a signal–background ratio of 1.5 or greater were considered to represent positive expression (+), and the others were considered to represent negative expression (−). A miRNA was considered to be differentially expressed only if it exhibited different expression between SW480 and SW620 cells.

### miRNA target prediction

The targets of miR-10a were predicted using the TargetScan, PicTar, miRanda algorithms in combination with their related functions.

### Quantitative real-time PCR

The stem-loop RT-qPCR analysis for the detection of miRNA level was performed using a Hairpin-it miRNAs RT-PCR Quantitation Kit (GenePharma, Suzhou, China). U6 was used to normalize the miR-10a expression. The RT-PCR analyses for the detection of MMP14 and ACTG1 in CRC cells and tissues were performed using SYBR Premix Ex Taq (TaKaRa, Otsu, Shiga, Japan) and a PikoReal 96 RT-PCR System (Thermo Fisher, Vantaa, Finland), *β*-actin was used to normalize the expression of MMP14 and ACTG1. The primer sequences are provided in Supplementary Table S2. All experiments were carried out at least in triplicate.

### Cell viability assay

Cells were seeded in 96-well plates at a density of 7 × 10^3^ per well in the corresponding growth medium. After 24 h, a Cell Counting Kit-8 (CCK-8, Dojindo Molecular Technologies, Shanghai, China) assay was performed according to the manufacturer's instructions. The absorbance at a wavelength^38^ of 450 nanometers was detected using a Thermo Scientific Mulyiskan FC Microplate Spectrophotometer (Shanghai, China). Each experiment was carried out in three replicate wells and was repeated three times.

### Boyden chamber transwell migration or invasion assay

A transwell chamber culture system (8 *μ*m pore, Corning, NY, USA) was used to detect the migration or invasion capability of cells. Cells (1 × 10^5^ per well) were seeded in Boyden chamber transwells without or with Matrigel-coated inserts with serum-free growth medium. Complete growth medium containing 10% fetal bovine serum was added to the lower chamber. After 24 and 48 h, the cells attached to the lower surface of the insert filter were fixed with 33% (v/v) acetic acid (glacial acetic acid:methyl alcohol is 1 : 3) and stained with 5% crystal violet and counted. For the invasion assay, the upper chambers were coated with 40 *μ*l at a concentration of 2 mg/ml of Matrigel (BD Biosciences, Bedford, MA, USA) for 1 h at 37 °C, and the time for invasion is 72 h. For the modified migration assay, the lower chamber was added with growth medium containing 10% fetal bovine serum supplemented with TGF-*β* (100 mg/l, PeproTech, Rocky Hill, USA) to induce migration of the CRC cells.^41, 42^

### Cell–cell adhesion assay

Cells were washed with calcium/magnesium-free phosphate-buffered saline and detached from the culture dishes with 4 mM EDTA in calcium/magnesium-free phosphate-buffered saline to preserve the cell surface expression of cadherin subtypes. The cells were separated into a single-cell suspension with a Pasteur pipette. After centrifugation, the cells were resuspended at a final concentration of 5 × 10^5^ cells/ml in calcium-free, suspension-modified Eagle's medium in the absence of serum and with or without cell adhesion inhibitor peptide (cyclic RGDfv, 100 *μ*M, Nanjing Peptide Biotech Ltd, Nanjing, China), then 1 × 10^6^ cells were maintained in suspension on 10% poly-2-hydroxyethyl methacrylate-coated (poly-HEMA, Sigma, St. Louis, MO, USA) six-well plates to prevent cell attachment to the substrate. Cells on the substrate in at least three fields per well were imaged after 24 h of culture using an Olympus IX 71 (Tokyo, Japan). Cell aggregates were counted based on the number of cells per aggregate: 24 h (>20 cells).

### Cell–matrix adhesion assay

In all, 96-well plates were coated overnight with 10 *μ*g/ml FN (Solarbio, Shanghai, China) and 200 *μ*g/ml Matrigel at 4 °C and were blocked with 1% (w/v) bovine serum albumin. Cells were suspended in complete medium with or without RGDfv (100 *μ*M) and seeded on the 96-well plates at a density of 2 × 10^4^ per well in triplicate, allowed to adhere at 37 °C for at least 10 min (min) and were then washed three times with phosphate-buffered saline. The cells were fixed with 4% (w/v) paraformaldehyde, stained with 0.5% (w/v) crystal violet for 10 min, and then the attached cells lysed with 30% (v/v) glacial acetic acid for 15 min; absorbance at 620 nm was then measured.^20, 43^ For analysis of SW480 and SW620 cells, the adherent cells were measured by software of ImageJ (National Institutes of Health, MD, USA).

### FACS assay

Flow cytometry was used to analyze DNA degradation in response to the disruption of cell–matrix interactions. Cells (2 × 10^5^) were incubated on tissue culture or poly (2-hydroxyethyl methacrylate)-coated 100-mm dishes for 12 or 48 h, collected and stained with Annexin V-FITC/PI (KGA, Nanjing, China). The apoptotic cells were analyzed using a BD Aria II (San Jose, CA, USA).

### *In vivo* metastasis assay

Nude mice were randomly assigned to two groups (*n*=5), anesthetized with isoflurane and subjected to laparotomy. The spleen was delivered through a 1-cm incision in the upper left lateral abdomen, and 1 × 10^6^ cells (SW620-pri-miR-10a or SW620-Ctrl) resuspended in 25 *μ*l of minimum essential medium-a and mixed with 25 *μ*l of Matrigel (1 mg/ml) were injected into the distal tip of the spleen. The spleen was then replaced in the abdomen, and the abdominal cavity was closed with staples. After 6–8 weeks, the mice were killed, and the spleens and livers were removed for pathological examination. Transverse sections (5 mm) of the liver were prepared at 10 different levels to cover the entire liver, and the sections were stained with H&E. Metastatic nodules were counted in a double-blind manner under microscopy as previously described.^44^

### Vector construction

To construct an EGFP reporter vector, the EGFP coding region from pEGFP-N2 (Clontech, Mountain View, CA, USA) was amplified by PCR. The amplified fragment was cloned into pcDNA3 at the *Hind*III and *Bam*HI sites to form the pEGFP vector. To construct the reporter vectors containing the binding sites of miR-10a to ACTG1/MMP14 3′-UTR, PCR was performed to amplify the fragments using the following primers: ACTG1 forward, 5′-GTGGATCCTTTGCTGCATGGGTTA-3′ ACTG1 reverse, 5′-CTGAATTCTACGGCTTGGACTTTC-3′ MMP14 forward, 5′-CGCGGATCCAGAACCTTGCCCAAACTCAG-3′ and MMP14 reverse, 5′-GAGGAATTCGTGAGACAGGCTTGAGG-3′. The amplified fragments were cloned into pEGFP at the *Bam*HI and *Eco*RI sites located just downstream of the EGFP coding region. The constructed vectors were named pEGFP/ACTG1 3′-UTR and pEGFP/MMP14 3′-UTR. Similarly, the fragments of the ACTG1 3′-UTR and MMP14 3′-UTR mutants, which contained the mutated binding sites of miR-10a, were cloned into the pEGFP at the same sites. The constructed vectors were named pEGFP/ACTG1-M and pEGFP/MMP14-M. To construct the miR-10a expression vector, a 436-bp fragment containing the miR-10a precursor sequence (mimic of pri-miR-10a) was amplified from human genomic DNA by PCR using the following primers: pri-miR-10a forward, 5′-CATTCGGATCCCAAGAACAGACTCGCAC-3′ and pri-miR-10a reverse, 5′-GGGAGAATTCGGGGAGAGTTCAGGTAGATG-3′. The amplified fragment was cloned into pcDNA3 at the *Bam*HI and *Eco*RI sites to form the pcDNA3/pri-miR-10a vector.

### Fluorescent reporter assay

Cells were seeded in 24-well plates the day before transfection. The cells were first transfected with oligonucleotides or pri-miR-10a expression vectors and were then transfected with the EGFP reporter vectors on the next day. The red fluorescence protein expression vector pDsRed2-N1 (Clontech) was spiked in and used for normalization. The cells were lysed with radioimmunoprecipitation assay lysis buffer 72 h after transfection, and the proteins were harvested. The intensity of EGFP and red fluorescence protein was detected with a Fluorescence Spectrophotometer F-4500 (HITACHI, Tokyo, Japan).

### Western blot

For the western blot analysis, protein samples were obtained by lysing CRC cells in standard sample buffer (50 mM Tris-HCl, pH 6.8, 2% SDS, 10% glycerol) for SDS-PAGE. The following primary antibodies were used at the indicated dilutions: anti-E-cadherin pAb and anti-vimentin (1 : 2000, Saier, Tianjin, China); anti-Bcl-2 pAb and anti-GAPDH pAb (1 : 2000, Saier) and anti-*β*-integrin pAb (1 : 1000, Abcam, Cambridge, MA, USA). Optical band density was quantified using LANE 1D champgel 5000 (Beijing, China) software.

### IHC

FFPE CRC tissues were sectioned to a thickness of 5 *μ*m. After routine deparaffinization, rehydration, blocking with hydrogen peroxide and tissue antigen retrieval with a microwave for 80 s, the samples were incubated with rabbit anti-MMP14 and anti-ACTG1 polyclonal antibodies (SRP00262, SRP08114, 1:300, Saier) overnight at 4 °C. The slides were stained with secondary antibodies and diaminobenzidine tetrahydrochloride (ZSGBBIO, Beijing, China), and then counterstained with hematoxylin. The stained slides were evaluated independently by two pathologists who were unaware of the clinical parameters.

### Statistical analysis

*In vitro* data obtained from three independent experiments were analyzed using Student's *t*-test. Significant associations between miR-10a expression and clinicopathological parameters were assessed using the Pearson correlation. *P<0.05, **P<0.01 and ***P<0.001 were considered significant. The mean±S.D. of three or more independent experiments is reported.
