# Supplementary material for: miR-10a suppresses colorectal cancer metastasis by modulating the epithelial-to-mesenchymal transition and anoikis
Source: Cell Death Dis. 2017 Apr 6;8(4):e2739–. doi: 10.1038/cddis.2017.61 (PMC5477594; doi:10.1038/cddis.2017.61)
Supplement: Supplementary Information [file cddis201761x1.doc]

**Supplementary information**

**miR-10a suppresses colorectal cancer metastasis by modulating the epithelial-to-mesenchymal transition and anoikis**

Yankun Liu^1,2^, Yingnan Zhang^1^, Haidong Wu^1^, Yufeng Li^2^, Yi Zhang^1^, Min Liu^1^, Xin Li^1^, Hua Tang^1^

^1^Tianjin Life Science Research Center, Department of Pathogen Biology, School of Basic Medical Sciences, Tianjin Medical University, Tianjin 300070, China

^2^ The Cancer Institute, Tangshan People’s Hospital, Tangshan 063001, China

Running title: miR-10a inhibits colorectal cancer metastasis

Correspondence: Hua Tang, E-mail: [htang2002@yahoo.com](mailto:htang2002@yahoo.com); tangh@tmu.edu.cn

**Supplementary tables**

Table S1 Correlation of miR-10a Expression in Tissues with Clinical CRC Patient Pathological Characteristics

|  | All cases | miR-10a expression |  |  |
| --- | --- | --- | --- | --- |
| Variables | (N=26) | (primary-metastasis)/primary | *P* values ^a^ | *Pearson* |
| Age, y |  |  | 0.746 | 0.067 |
| ≤65 ^b^ | 15 | 0.59 ± 0.25 |  |  |
| >65 | 11 | 0.51 ± 0.30 |  |  |
| Sex |  |  | 0.672 | -0.087 |
| Male | 13 | 0.53 ± 0.25 |  |  |
| Female | 13 | 0.57 ± 0.30 |  |  |
| Tumor site ^c^ |  |  | 0.674 | -0.087 |
| Proximal colon | 3 | 0.59 ± 0.35 |  |  |
| Distal colon | 14 | 0.58 ± 0.28 |  |  |
| Rectal colon | 9 | 0.51 ± 0.25 |  |  |
| Tumor type |  |  | 0.377 | 0.057 |
| Adenocarcinoma | 22 | 0.57 ± 0.30 |  |  |
| Mucinous*^e^* | 4 | 0.47 ± 0.33 |  |  |
| CEA level, ng/ml |  |  | 0.245 | -0.237 |
| 0-5 | 16 | 0.66 ± 0.26 |  |  |
| >5 | 10 | 0.39 ± 0.18 |  |  |
| CA199 level, U/ml |  |  | 0.813 | 0.049 |
| 0-35 | 20 | 0.55 ± 0.27 |  |  |
| >35 | 6 | 0.56 ± 0.27 |  |  |
| CA724 level, U/ml |  |  | 0.896 | 0.027 |
| 0-6.9 | 17 | 0.56 ± 0.27 |  |  |
| >6.9 | 9 | 0.55 ± 0.28 |  |  |
| Invasion depth |  |  | 0.012 ^d^ | -0.483 |
| T1+T2 | 12 | 0.71 ± 0.27 |  |  |
| T3+T4 | 14 | 0.43 ± 0.19 |  |  |
| No. of positive nodes |  |  | 0.706 | 0.078 |
| 0 | 0 | 0 |  |  |
| 1-4 | 18 | 0.50 ± 0.26 |  |  |
| 5+ | 8 | 0.57 ± 0.30 |  |  |
| Distant metastasis |  |  | 0.015 ^d^ | -0.474 |
| M0 | 22 | 0.77 ± 0.08 |  |  |
| M1 | 4 | 0.60 ± 0.25 |  |  |

CEA, carcinoembryonic antigen; CA199, carbohydrate antigen 19-9; CA724, carbohydrate antigen 72-4.

^a^Pearson bivariate analysis.

*^b^*Median age at operation.

*^c^*Proximal colon tumors are those arising in the cecum, ascending colon, hepatic flexure, or transverse colon; distal colon tumors are those arising in the splenic flexure, descending colon, or sigmoid colon; and rectal tumors are those arising in the rectosigmoid or rectum.

*^d^*Statistically significant (^*^*P*<0.05).

*^e^*The mucinous type includes mucinous adenocarcinoma and signet ring cell carcinoma.

| Table S2 Primer Sequences used for Real-Time PCR | | | | | |
| --- | --- | --- | --- | --- | --- |
| Gene | Primer | Sequence (5’; 3’) | Length   (bp) | T (°C) | Cycles |
| miR-10a | RT  FO | GTCGTATCCAGTGCAGGGTCCGAGGTATTCGCACTGGATACGACCACAAATTC  TGCGGTACCCTGTAGATCCG |  |  |  |
|  | RE | CCAGTGCAGGGTCCGAGGT |  | 62 | 40 |
| miR-10a siRNA |  | CACAAAUUCGGAUCUACAGGGUA |  |  |  |
| miR-10a NC |  | CAGUACUUUUGUGUAGUACAA |  |  |  |
| MMP14 siRNA 1# |  | CCAGGGUCUCAAAUGGCAATT |  |  |  |
|  |  | UUGCCAUUUGAGACCCUGGTT |  |  |  |
| MMP14 siRNA 2# |  | GCGAUGAAGUCUUCACUUATT |  |  |  |
|  |  | UAAGUGAAGACUUCAUCGCTT |  |  |  |
| ACTG1 siRNA 1# |  | GACAGGAUGCAGAAGGAGATT |  |  |  |
|  |  | UCUCCUUCUGCAUCCUGUCTT |  |  |  |
| ACTG1 siRNA 2# |  | CCAGCACCAUGAAGAUCAATT |  |  |  |
|  |  | UUGAUCUUCAUGGUGCUGGTT |  |  |  |
| NC FAM |  | UUCUCCGAACGUGUCACGUTT |  |  |  |
|  |  | ACGUGACACGUUCGGAGAATT |  |  |  |
| MMP14 (clone CDS) | FO | CGCGGATCCAGAACCTTGCCCAAACTCAG | 238 | 56 | 35 |
|  | RE | CGCGAATTCGGACAGGGACCAACAGGAG |  |  |  |
| ACTG1 (clone CDS) | FO | CTGAATTCTACGGCTTGGACTTTC | 458 | Gradient  PCR^a^ | 31 |
|  | RE | GTGGATCCTTTGCTGCATGGGTTA |  |  |  |
| β-actin | FO | CGTGACATTAAGGAGAAGCTG | 499 | 58 | 35 |
|  | RE | CTAGAAGCATTTGCGGTGGAC |  |  |  |

T, temperature; mut, mutation type; RT, reverse transcriptase; NC, negative control. ^a^Conditions: 94 °C for 3 min; 94 °C for 1 min, 50-65 °C (-0.5 °C) for 30 s, and 72 °C for 1 min for 31 cycles; 72 °C for 10 min; 4 °C indefinitely.

**Supplementary Figures**


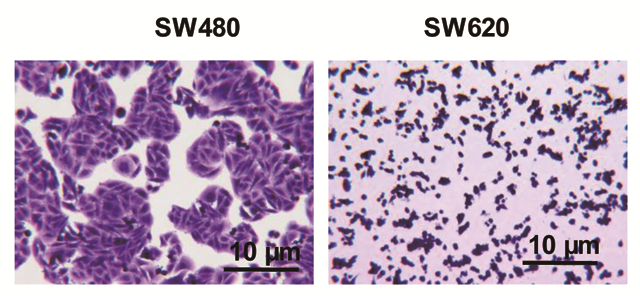


Figure S1. SW480 cells with an elongated morphology and SW620 cells with a rounded shape for cultures. The images of SW480 and SW620 cells before seeded into the 96 well plates: SW480 and SW620 cells were seeded in the 24-well plate with 5×10^4^ cells per well, 48 h later cells were stained with 0.5% (w/v) crystal violet，the photomicrographs were taken at 100× magnification


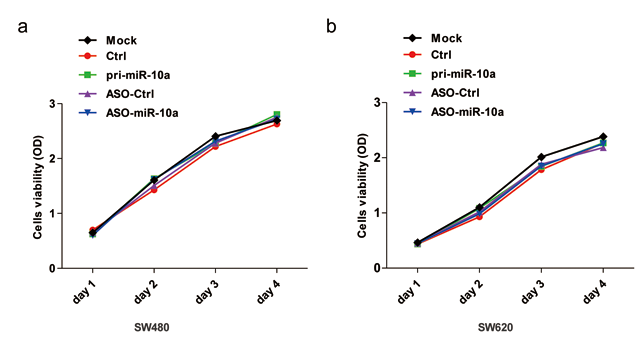


Figure S2. The altered levels of miR-10a did not significantly affect SW480 or SW620 cell viability and proliferation. (**a** and **b**) CCK-8 assay of SW480 cells and SW620 cells transfected with ASO-miR-10a and pri-miR-10a compared with the control groups for 4 days, respectively. *P*>0.05


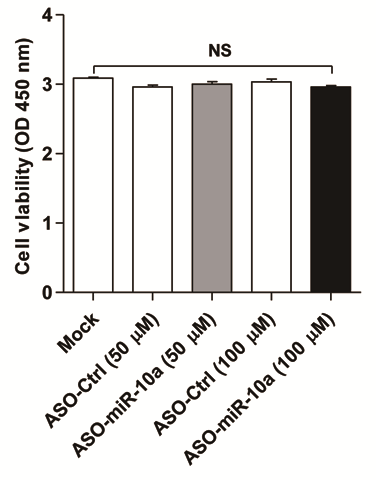


Figure S3. miR-10a silencing did not altered the cell viaibily. The absorbance of SW480 cells transfected with different concentrations of ASO-miR-10a suspension-cultured for 48 h at 450 nm wavelength. (NS, not significant)


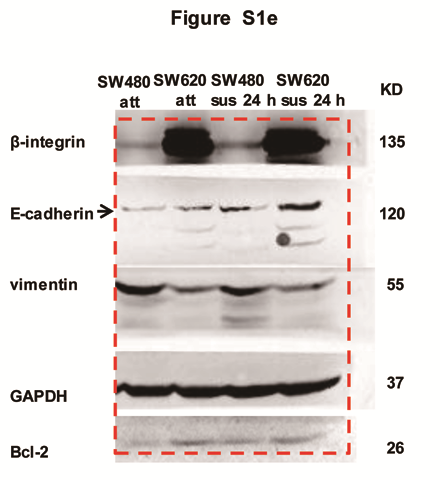


Figure S4. Uncropped, unprocessed image of blots and gels for Figure 1e.


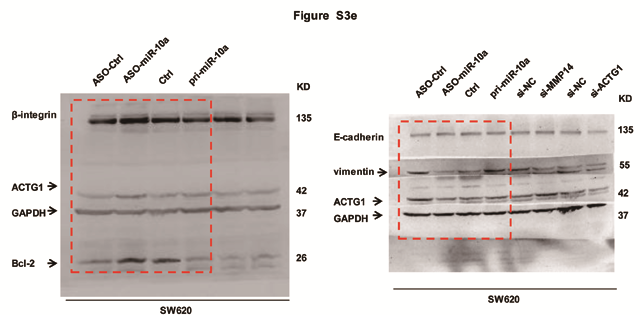


Figure S5. Uncropped, unprocessed images of blots and gels for Figure 3e.


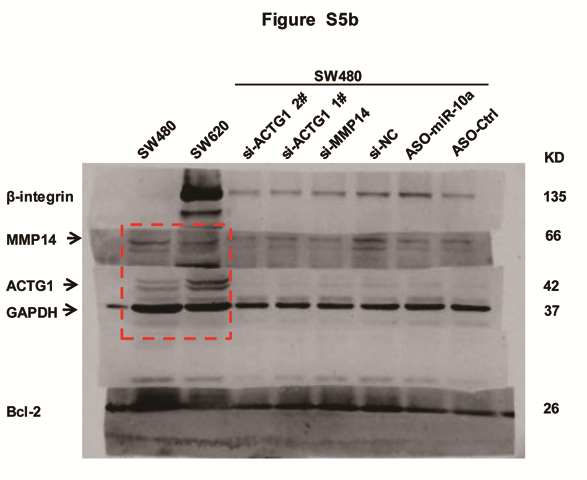


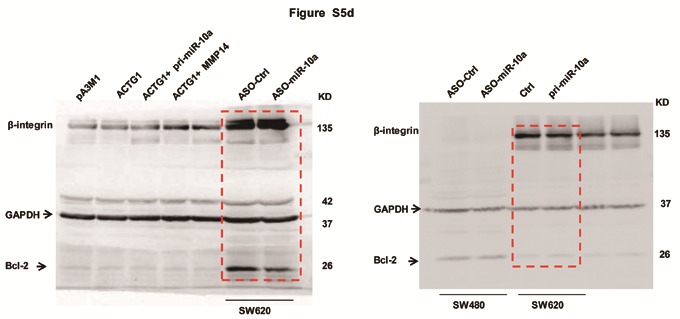


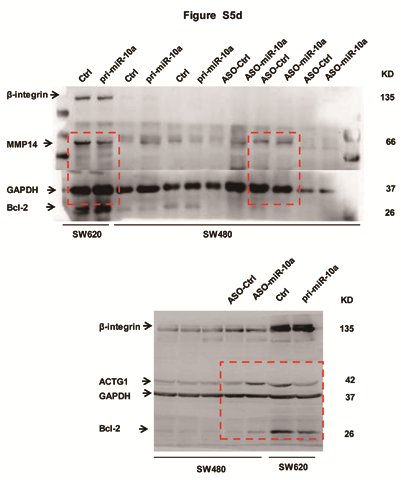


Figure S6. Uncropped, unprocessed images of blots and gels for Figure 5b and 5d.


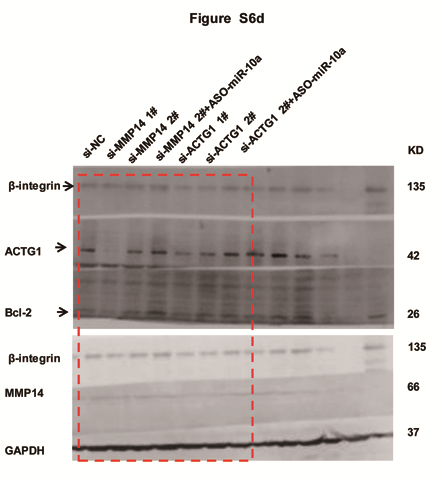


Figure S7. Uncropped, unprocessed image of blots and gels for Figure 6d.


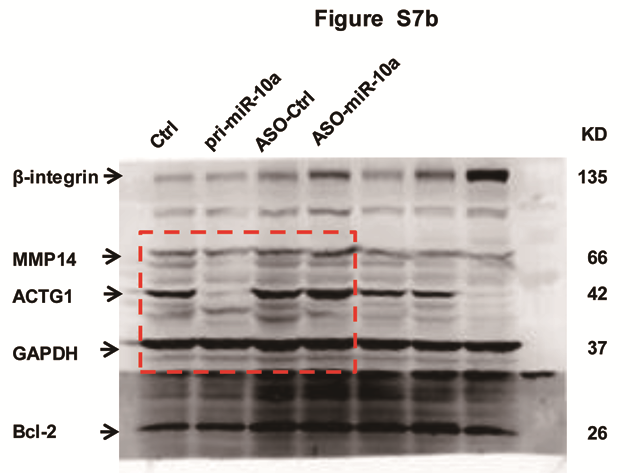


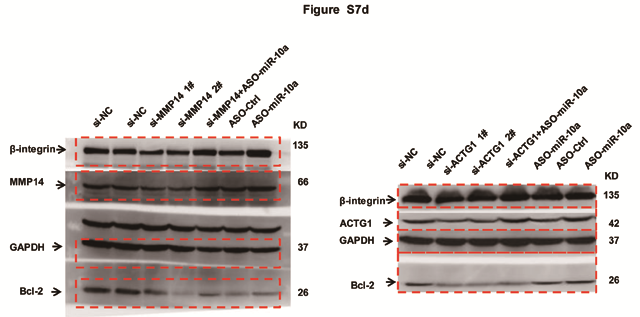


Figure S8. Uncropped, unprocessed images of blots and gels for Figure 7b and 7d.
